# Tailoring the Lithium Concentration in Thin Lithium Ferrite Films Obtained by Dual Ion Beam Sputtering

**DOI:** 10.3390/nano14141220

**Published:** 2024-07-18

**Authors:** Pilar Prieto, Cayetano Hernández-Gómez, Sara Román-Sánchez, Marina París-Ogáyar, Giulio Gorni, José Emilio Prieto, Aida Serrano

**Affiliations:** 1 Departamento de Física Aplicada M-12, Universidad Autónoma de Madrid, 28049 Madrid, Spain; cayetano.hernandez@uam.es; 2 Instituto Nicolás Cabrera (INC), Universidad Autónoma de Madrid, 28049 Madrid, Spain; 3 Departamento de Electrocerámica, Instituto de Cerámica y Vidrio (ICV), CSIC, 28049 Madrid, Spain; sara.roman@icv.csic.es (S.R.-S.); marina.paris@uam.es (M.P.-O.); aida.serrano@icv.csic.es (A.S.); 4 Departamento de Física de Materiales, Universidad Autónoma de Madrid, 28049 Madrid, Spain; 5 Laser Processing Group, Institute of Optics, CSIC, C/Serrano 121, 28006 Madrid, Spain; g.gorni@csic.es; 6 Alba Synchrotron Light Facility, CELLS, 08290 Barcelona, Spain; 7 Instituto de Química Física Blas Cabrera (IQF), CSIC, C/Serrano 119, 28006 Madrid, Spain; jprieto@iqf.csic.es

**Keywords:** lithium ferrite, lithium concentration, ion beam assistance, X-ray absorption spectroscopy, Raman microscopy

## Abstract

Thin films of lithium spinel ferrite, LiFe_5_O_8_, have attracted much scientific attention because of their potential for efficient excitation, the manipulation and propagation of spin currents due to their insulating character, high-saturation magnetization, and Curie temperature, as well as their ultra-low damping value. In addition, LiFe_5_O_8_ is currently one of the most interesting materials in terms of developing spintronic devices based on the ionic control of magnetism, for which it is crucial to control the lithium’s atomic content. In this work, we demonstrate that dual ion beam sputtering is a suitable technique to tailor the lithium content of thin films of lithium ferrite (LFO) by using the different energies of the assisting ion beam formed by Ar^+^ and O_2_^+^ ions during the growth process. Without assistance, a disordered rock-salt LFO phase (i.e., LiFeO_2_) can be identified as the principal phase. Under beam assistance, highly out-of-plane-oriented (111) thin LFO films have been obtained on (0001) Al_2_O_3_ substrates with a disordered spinel structure as the main phase and with lithium concentrations higher and lower than the stoichiometric spinel phase, i.e., LiFe_5_O_8_. After post-annealing of the films at 1025 K, a highly ordered ferromagnetic spinel LFO phase was found when the lithium concentration was higher than the stoichiometric value. With lower lithium contents, the antiferromagnetic hematite (α-Fe_2_O_3_) phase emerged and coexisted in films with the ferromagnetic Li_x_Fe_6-x_O_8_. These results open up the possibility of controlling the properties of thin lithium ferrite-based films to enable their use in advanced spintronic devices.

## 1. Introduction

The ability to generate pure spin currents in a ferromagnetic insulator allows the propagation of information via the spin without a charge current and its associated losses, thus potentially improving energy efficiency in spin-based computing and memory. Y_3_F_e5_O_12_ (YIG) is currently the most promising material, one in which the spin waves can carry information over distances as large as millimeters with frequencies up to the THz regime, due to its low damping constant. However, the growth of high-quality thin YIG films requires single-crystal garnet substrates and high processing temperatures [1,2], which makes their integration with existing technology a challenge. Alternative materials, which are more easily integrable in real spintronic devices, include some classes of spinel ferrites satisfying the main requirements regarding the origin of the low damping constant, i.e., structural order and minimum orbital momentum [3]. By mimicking the cation chemistry of YIG, in which the orbital angular momentum of all the cations is zero (L = 0), with the valence shells of magnetic Fe^3+^ being half-filled and those of nonmagnetic Y^3+^ completely filled, it has been demonstrated that it is possible to obtain an ultra-low damping constant in thin spinel ferrite films, as, for example, on MgAl_0.5_Fe_1.5_O_4_, which exhibits ultralow magnetic damping that is enabled by its L = 0 cation chemistry and high-quality coherent epitaxy. Its magnetism arises from Fe^3+^, while the Mg^2+^ and Al^3+^ cations have zero orbital angular momentum because of their full valence shells [3,4]. This is also the case with the reported ultra-low magnetic damping of thin LiFe_5_O_8_ films grown on MgAl_2_O_4_ substrates [5]. The mechanism that governs their low damping activity is the presence of the half-filled Fe^3+^L = 0 3d^5^ electron configuration, as the primary magnetic ion and the non-magnetic Li^+^ have zero orbital angular momentum, which is related to the suppression of spin-orbit interactions that reduce coupling between the spins and the lattice.

In addition, LiFe_5_O_8_ has also drawn special attention because of its excellent dielectric and magnetic properties, such as its high Curie temperature (~950 K), high electric resistivity, high saturation magnetization and strong magnetocrystalline anisotropy, low losses at high frequencies, and good chemical and thermal stability [6,7,8]. These characteristics give it the potential for application in various components, not only in microwave devices and spintronics [9], but it has also attracted interest for gas sensing and as a low-cost cathode active material for rechargeable Li-ion batteries [10,11]. In fact, it has recently been demonstrated that it is also a magnetoelectric material with a high coupling temperature [12]. Other interesting applications of thin LFO films are due to the possibility of controlling magnetism by lithium insertion, as has been proven when it is used as an electrode in secondary battery cells [13]. Reizt et al. demonstrate that Li insertion into LFO allows the tailoring of the bulk magnetic state in a highly reversible manner; this magnetoionic control is one of the many possibilities that are being explored regarding the development of new spintronic devices based on the control of magnetism by electric fields [14].

LiFe_5_O_8_ has an inverse spinel structure, where the tetrahedral sites are occupied by Fe^3+^, and the octahedral sites are shared by Li^+^ and the remaining Fe^3+^ at a ratio of 1:3 (denoted as Fe[Li_0.5_Fe_1.5_]O_4_). The antiparallel-aligned magnetic spin between the Fe^3+^ distributed at tetrahedral sites and octahedral sites causes ferrimagnetic features and leads to a high magnetic moment of 2.5 μB per formula unit [15], indicating that the atomic occupancy is very important for determining the magnetic behavior of thin LFO films. Specifically, there exist two polymorphic forms of LiFe_5_O_8_: the ordered α-phase (space group P4_3_32), which is characterized by the presence of the 1:3 ordering of Li^+^ and Fe^3+^ ions on octahedral sites, and the β- disordered phase (space group Fd-3m) in which these ions are distributed randomly [15]. At temperatures above 993–1023 K, the ordering is destroyed, and the Li and Fe are distributed randomly over the octahedral sites in an order-disorder transition [15,16]. In addition, the introduction of Li defects into the LFO spinel structure could change its magnetic behavior, not only due to the possible presence of Fe^2+^ but also due to inducing an increase in the lattice constant [17,18]. In fact, it is expected that a material with less than the stoichiometric Li content will have a mixture of Fe^2+^ and Fe^3+^ on its octahedral sites and may exhibit semiconducting behavior [17].

The deposition of single-phase LiFe_5_O_8_ films is quite difficult to assess, due not only to the volatility of Li and the oxygen loss during the deposition process but also due to the formation of the impurity phases of LiFeO_2_ and Fe_2_O_3_ during the post-annealing process. For this reason, the most suitable deposition method to obtain thin stoichiometric LiFe_5_O_8_ films is with pulsed laser deposition (PLD) [5,7,14,17,18,19,20]. However, other deposition techniques, such as high-pressure sputtering [21,22], chemical vapor deposition (CVD) [23], and sol-gel dip coating [13] have also been used.

LFO is cubic in form, with a lattice constant nearly twice as large as that of MgO; thus, LFO grows epitaxially on MgO substrates [6,18,23]. Other cubic substrates such as MgAl_2_O_4_ [5,6,21] and SrTiO_3_ [6,24], in which the growth of LFO is isostructural, have also been used. Whereas LFO on MgO grows under compressive stress, on MgAl_2_O_4_ and SrTiO_3_ substrates, growth occurs under tensile stress. In addition, highly textured (111) thin LFO films have also been grown on (0001) Al_2_O_3_ substrates [17,19,20].

The tuning of the physical properties of magnetic thin films as LFO is an interesting topic for the development of spintronic devices, which can be controlled by using external electric fields [14], by the interfaces and thickness of the film [21], and, of course, by deposition parameters such as, for example, the temperature during the growth process [18]. Other approaches to tuning and improving the physical characteristics of LFO involve Mg or Al substitution of the Fe atoms [4,25,26,27]. For example, it has been demonstrated that the degree of Al substitution on LFO films tunes its saturation magnetization and in-plane magnetic anisotropy, thereby keeping excellent crystalline quality as well as low magnetic damping [27]. Another possibility is through the introduction of Li or Fe vacancies into the LFO lattice; however, due to difficulties in the quantification of Li, the impact of Li concentration on the properties is less well studied.

In this work, we propose an alternative method to obtain stoichiometric and highly (111) oriented thin LFO films by the dual ion beam sputtering technique, in which the energy of the assisting ion beam is the main parameter used to control the phase and the amount of Li in the LFO structure. Deep characterization, based on a combination of X-ray diffraction (XRD), Rutherford backscattering spectroscopy combined with nuclear reaction analysis (RBS-NRA), X-ray absorption near-edge structure (XANES), extended X-ray absorption fine structure (EXAFS), and confocal Raman micro-spectroscopy has allowed us to clarify the relationship between the Li content and the compositional and structural properties of thin LFO films.

## 2. Materials and Methods

Thin lithium ferrite films were deposited on (0001) Al_2_O_3_ substrates by dual ion beam sputtering (DIBS): ion beam sputtering is emitted from a LiFeO_2_ target using Ar^+^ ions from a 3 cm Kaufmann-type ion source under simultaneous bombardment of the deposited layer, with a controlled mixture of low-energy oxygen and argon ions from an end-Hall ion source. The base pressure of the vacuum chamber was 2 × 10^−5^ Pa, while the pressure during the growth process was 4 × 10^−2^ Pa. The substrate temperature of 825 K, the Ar^+^ sputtering ion energy of 800 eV, and a current density of 2.4 mA/cm^2^ were kept constant for all the samples. The current density of the assisting ion beam was also kept constant at 0.065 mA/cm^2^, while the energy of the assisting beam formed by 70% of Ar^+^ and 30% O_2_^+^ ions varied between 37 and 70 eV. A thin film without assistance was also prepared with the same oxygen partial pressure, i.e., P_O2_ = 2.5 × 10^−2^ Pa. During the deposition, the substrates rotated at 2 rpm to increase the homogeneity of the deposited material. The films were also post-annealed at 1025 K for 10 min in air and both the as-deposited and post-annealed thin films have been studied.

The elemental composition of the samples (Li, Fe, O) was evaluated by means of ion beam analysis techniques. RBS, combined with NRA, was carried out with the 5 MV tandem accelerator at CMAM [28], using H^+^ at 3.0 MeV, while RBS in random and channeling configurations was performed with ^4^He^+^ at 1.8 MeV. A silicon barrier detector was used to measure the backscattering yield and the sample position was controlled with a three-axis goniometer. The experimental conditions for the RBS-NRA measurements were specifically chosen to detect and quantify the Li that was present in the sample, in which a scattering angle of 150° was used. Non-invasive characterization of the lithium content was performed using the ^7^Li(p,α)^4^He nuclear reaction, the cross-section of which shows a broad maximum at a proton energy of 3 MeV [29]. This nuclear reaction is considered the most suitable for lithium quantification since the signal intensity is proportional to the amount of the naturally occurring ^7^Li isotope, while yielding a high signal-to-noise ratio [29,30,31]. The in-depth quantification and distribution of Li and all the other elements in the samples were determined with the SIMNRA simulation software package [32].

The crystal structure and texture of the different films were analyzed by X-ray diffraction (XRD) in a θ/2θ configuration, using a PANanalytical X’Pert MPD system with Cu-Kα radiation.

X-ray absorption spectroscopy (XAS) experiments were carried out on the LFO films at the BL22 CLÆSS beamline of the ALBA synchrotron facility in Cerdanyola del Vallès (Spain) [33]. Both XANES and EXAFS experiments were carried out at room temperature in fluorescence mode at the Fe K-edge (7112 eV). A monochromator of double Si crystal oriented in the (311) direction was employed, using an Fe metal foil to calibrate the energy. Several oxide references, such as LiFeO_2_, LiFe_5_O_8_, α-Fe_2_O_3_, γ-Fe_2_O_3_, and Fe_3_O_4_, were also measured in this research in transmission mode. The analysis of absorption spectra was conducted using the Athena and Artemis programs [34].

Confocal micro-Raman experiments were also carried out on the thin films. The measurements were collected using a confocal Raman microscope (CRM) WITec ALPHA 300RA at room temperature with a Nd:YAG linearly polarized laser (532 nm). The Raman spectra were recorded in the range of 60–3600 cm^−1^, using an objective with a numerical aperture of 0.95 and a laser excitation power of 3 mW to avoid any damage to the films. The Raman signal was collected from the confocal plane of the film with the highest intensity, averaging several Raman spectra from different regions. In addition, a compositional microanalysis of the film’s surface was achieved by Raman intensity mapping.

## 3. Results and Discussion

### 3.1. Lithium Quantification

**Figure 1:**
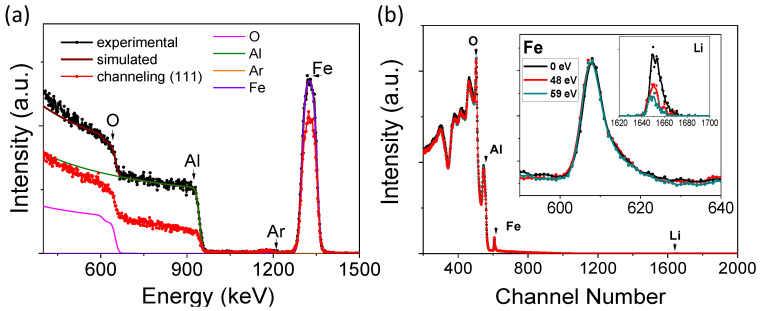
(**a**) Random and (**b**) (111)-aligned RBS spectra obtained with ^4^He+ ions at 1.8 MeV of a thin LFO film grown by assisted ion beam sputtering, with the energy of the assisting beam at 48 eV, and deposited on a (0001) Al_2_O_3_ substrate. The results of the simulation with the SIMNRA program, including the fitting signal for O, Al, Ar, and Fe, are also shown. (**b**) RBS-NRA spectra obtained with H+ ions at 3.0 MeV and at a scattering angle of 150° for a thin LFO film, obtained by ion beam sputtering without assistance and with an assisting beam energy of 48 and 59 eV. The Li signal is zoomed in in the inset.

Figure 1a shows the RBS spectra on the random and channeling configuration of a thin LFO film grown on a (0001) Al_2_O_3_ substrate obtained with the energy of an assisting beam formed by Ar^+^ + O_2_^+^ ions of 48 eV. The difference between both spectra can be used to determine the epitaxial character or crystalline quality of the deposited layers. In this case, the difference is small compared to other materials such as NiO that have also been grown using the same deposition technique, i.e., ion beam sputtering [35,36]. However, the presence of channeling is an indication of a high degree of orientation along the (0001) Al_2_O_3_ substrate direction. The thickness and composition of the thin film can be obtained by a simulation with the SIMNRA program, giving thicknesses of between 91 and 104 ± 2 nm for the samples studied in this work. The Fe/O ratios can also be simulated, giving values of between 0.48 and 0.63, depending on the lithium concentration. However, no signal corresponding to lithium is visible in the spectra and just the concentration is simulated to accurately fit the Fe and O parts of the spectra. However, it is also possible to determine the concentration of Ar in the assisted thin LFO films, due to the Ar^+^ ion beam sputtering deposition process, as well as the 70% Ar^+^ ions present in the assisting beam, giving values ranging from 0.6% for the non-assisted film to ≈1.2% for the assisted film.

The amount of lithium content on the films is difficult to determine by conventional analysis techniques, including RBS, due to its low atomic number and small size. If RBS is combined with NRA by using the ^7^Li(p,α)^4^He nuclear reaction, it is possible to measure the lithium distribution as a function of depth without the necessity for any special sample preparation in a non-destructive way. This experimental approach has already been used to determine the composition and thickness of thin films on Li-ion batteries in electrode materials, such as with LiNiVO_4_ [31] or LiFePO_4_ electrodes [30]. We also used it to determine the amount of lithium in ceramic LFO ferrites in powder form in a previous work [37].

Figure 1b shows the RBS-NRA spectra obtained with 3 MeV H^+^ ions for three thin LFO films grown by ion beam sputtering without assistance and under an assisting beam energy of 48 and 59 eV. The surface signals from O, Al, Fe, and Li are marked by arrows. The Li signal is normalized to the iron signal and has been magnified in the insets of Figure 1b. It is clear that there are differences in the Li concentrations of the different thin LFO films; however, simulation of the RBS-NRA spectra is highly complicated because, in addition to the ^7^Li(p,α)^4^He nuclear reaction, we must consider other reactions for the Al and O nuclei with protons at 3 MeV energy and at a scattering angle of 150°. For that reason, we have performed a comparative study of the different thin films in terms of the relative area of the Li signal with respect to the corresponding area of Fe atoms and comparing these with the same area ratios obtained for the stoichiometric LiFe_5_O_8_ and LiFeO_2_ targets.

In order to determine the possible diffusion or volatility of lithium during post-thermal annealing, we have also measured the lithium content on thin LFO films before and after different post-annealing events in a temperature range of between 875 and 1125 K). We did not find a dependence of the lithium concentration on the post-annealing temperature and the differences in the lithium concentration in the full temperature range are lower than 15%.

**Figure 2:**
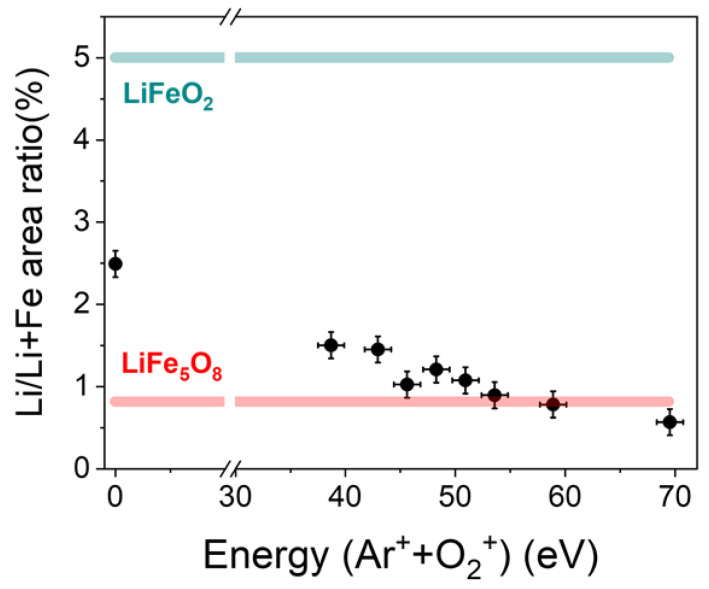
The Li/Li + Fe area ratio as a function of the energy of Ar^+^ + O_2_^+^ ions for thin LFO films without assistance (labeled as 0 eV) and ion-assisted films with energies ranging between 37 and 70 eV. The area ratio corresponding to a LiFe_5_O_8_ target and a LiFeO_2_ target have been included as red and green lines, respectively.

Figure 2 shows the lithium concentration in terms of the relative area, due to the Li atoms with respect to the total area and due to the Fe and Li atoms, compared with the corresponding value of the LiFe_5_O_8_ and LiFeO_2_ stoichiometric targets, which are marked as a red line and a green line, respectively, in Figure 2. A decrease in lithium concentration on the films is clearly visible as the energy of the ion beam assistance increases due to the preferential sputtering of the light elements, Li and O (Figure 2). However, the effect on oxygen is not observed because the assisting ion beam also contains 30% of O_2_^+^ ions. In dual ion beam sputtering processes, deposition parameters, such as the energy of the assisting ions, have a strong influence on the structure and composition of the deposited film, and, thus, on its physical properties [38]. Here, we demonstrate that by using the appropriate energy of the assisting ions, it is possible to obtain thin LFO films in a wide lithium concentration range, and, hence, tailor their physical properties.

### 3.2. Structural and Compositional Characterization

**Figure 3:**
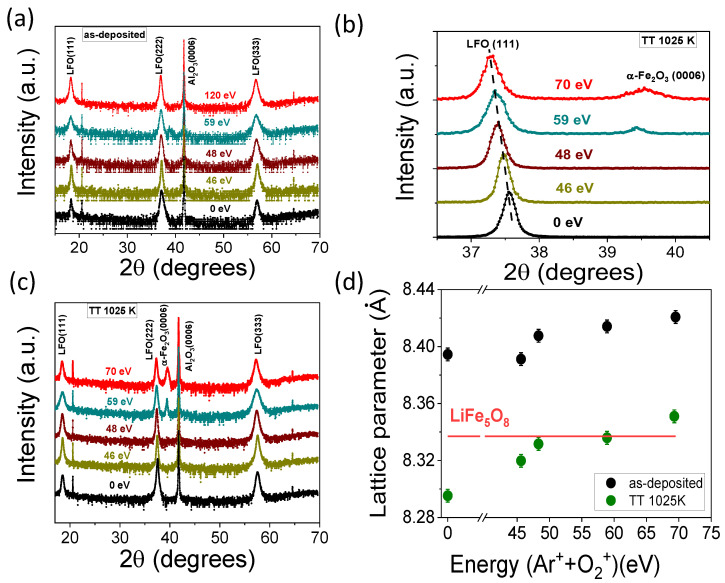
XRD diffraction pattern of the (**a**) as-deposited and (**b**) post-annealed thin LFO films obtained by dual ion beam sputtering. (**c**) The expanded region corresponding to the (222) LFO diffraction plane of the annealing samples. (**d**) The out-of-plane lattice parameter as a function of the energy of the assisting beam for the as-deposited thin LFO films and after post-thermal annealing.

Figure 3a shows the XRD pattern of thin LFO films obtained via DIBS with low-energy Ar^+^ + O_2_^+^ assisting ions on (0001) Al_2_O_3_ substrates. An intense peak can be distinguished, which is due to the (0006) Al_2_O_3_ diffraction plane as well as the (111), (222), and (333) Bragg’s peaks corresponding to a spinel cubic lithium ferrite structure. Thus, the films grow highly (111) out-of-plane-oriented without the presence of additional peaks, except for a small bump from (0006) hematite for the ion-assisted thin LFO film with an energy of 59 eV. Figure 3b shows the corresponding diffraction patterns of the post-annealed LFO films at 1025 K, also confirming the presence of a spinel structure and clearly identifying the (111), (222), and (333) diffraction peaks. In addition, the formation of the α-Fe_2_O_3_ phase can be observed on close-to-stoichiometric or lithium-deficient thin films, obtained when the assisting ion energy is higher than 54 eV. For the post-annealed thin LFO film, it is also clearly visible in Figure 3c that the (222) LFO peak moves to a lower angle, resulting in an increasingly out-of-plane lattice parameter as the energy of the assisting ion beam increases, which is similar to the trend found on the as-deposited films. Figure 3d shows this increase in lattice parameter for both the as-deposited and post-thermal-annealed films with the energy of the ion beam assistance that it is related with the loss of lithium from the lattice, as expected [18]. With respect to the as-deposited films, the post-annealed films exhibit lower values for the lattice parameter but these are closer to that of the bulk LiFe_5_O_8_ value, i.e., 8.337 Å (JCPDS card no. 17–115). The d_111_ values exceed the *a* lattice parameter of Al_2_O_3_ (i.e., a = 4.758 Å) by ≈2% in the as-deposited films and are reduced to ≈ 1% in the post-annealing samples.

**Figure 4:**
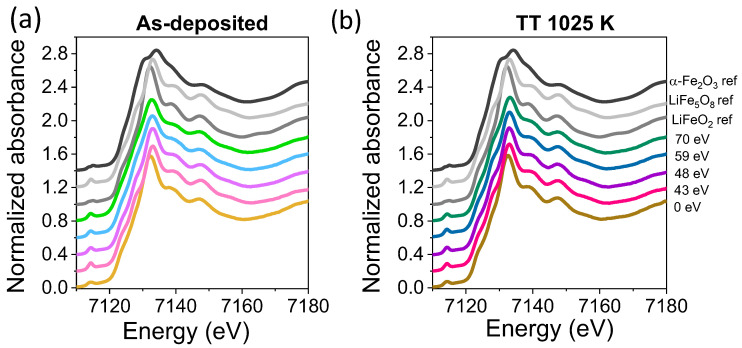
XANES spectra at the Fe K-edge for (**a**) as-deposited LFO films and (**b**) films after post-annealing treatment at 1025 K. XANES spectra for the references are also presented.

In order to corroborate the XRD results and to identify possible amorphous or long-range disordered phases, an XANES experiment was performed. Figure 4a,b shows the experimental XANES spectra at the Fe K-edge for as-deposited and post-annealed LFO films, respectively, varying the energy of the assisting ion beam. In all the as-deposited and post-annealed films, the absorption signal is very similar between them and that of the LiFe_5_O_8_ reference, except for films grown without ion assistance, which are more similar to the LiFeO_2_ reference and show similar characteristics at the absorption edge signal and at the position of the first resonance, shifted to lower values. These findings would indicate that the rock-salt phase of lithium ferrite identified herein by XANES in the sample prepared without assistance has a long-range disorder character, as it was not identified via XRD.

One of the main characteristics of the XANES spectra is the pre-edge peak. Its intensity depends on the oxidation state and site symmetry [39]. In octahedral coordination, the pre-edge peak of 3 d transition metals is primarily associated with weak 1 s to 3 d electric quadrupole transitions. When the coordination geometry deviates from perfect octahedral symmetry, this distortion can lead to p-d orbital mixing. This mixing enables stronger electric dipole transitions, as the p components of the hybridized p-d orbitals can interact with the core 1 s level, resulting in a more intense pre-edge peak. This is particularly evident in tetrahedral coordination, where the lack of inversion symmetry allows for significant p-d hybridization, leading to intense electric dipole transitions.

**Figure 5:**
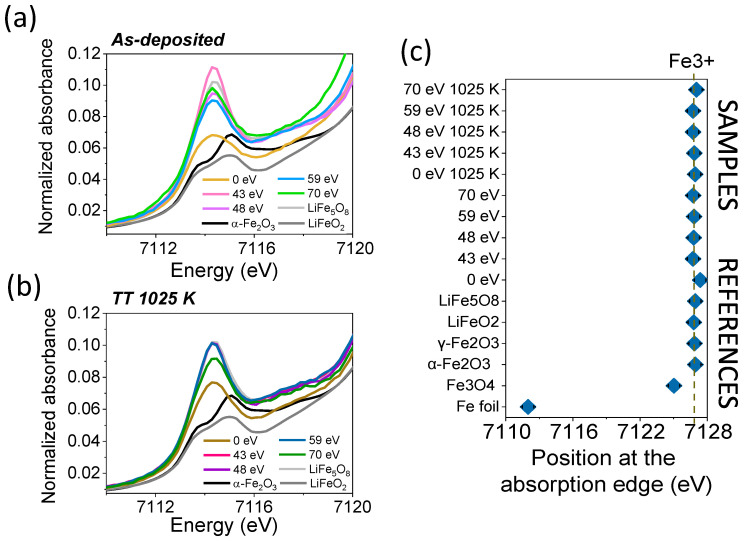
XANES pre-edge peak of (**a**) as-deposited LFO films and (**b**) films after a post-annealing at 1025 K. The pre-edge peak of the XANES spectra for the references is also presented. (**c**) Position at the absorption edge of XANES spectra at the Fe K-edge for as-deposited and post-annealed thin LFO films, as well as for the references, indicating the average oxidation state of Fe absorbing atoms in the samples.

The pre-edge region of the normalized XANES spectra is shown in Figure 5a,b for as-deposited and post-annealed films, respectively, along with the references. The pre-edge peak relating to the LiFeO_2_ reference exhibits a doublet peak structure that can be attributed to transitions to the t_2g_ and e_g_ states around 7113.7 eV and 7115.0 eV, respectively. The crystal field splitting is at 1.3 eV and the low intensity of the pre-edge transition is consistent with the octahedral coordination of the rock-salt structure. For the LiFe_5_O_8_ reference, there is a clear change in the pre-edge peak intensity and shape. A strong contribution is observed around 7114.2 eV that is related to the 1 s–3 d transitions of Fe^3+^ in the tetrahedral site, and a transition at 7116.5 eV associated with Fe^3+^ in the octahedral site is also noted [39].

The as-deposited films show the mixing of the pre-peak absorption signal between the pre-peak response of both lithium ferrites. For as-deposited films without assistance, the pre-peak is most similar to that of the LiFeO_2_ reference with a larger intensity, indicating some contribution by the LiFe_5_O_8_ reference. For the rest of the films prepared with ion assistance, the pre-peak signal is quite similar to that of the LiFe_5_O_8_ reference, with changes in the intensity of the peak at 7114.2 eV being related to the population of Fe^3+^ in tetrahedral positions. Specifically, the as-deposited film assisted with 59 eV Ar^+^ + O_2_^+^ ions shows the lowest pre-peak intensity, while that fabricated with 43 eV ions shows the most intense pre-peak, which is related to the highest tetrahedral coordination. For films after the post-annealing process, the pre-peak trend is similar to those of as-deposited films, with characteristics more similar to those of the LiFe_5_O_8_ reference. The lowest intensity is seen for the post-annealed film without assistance, indicating the lowest tetrahedral coordination, followed by the film assisted with 70 eV ions and by the rest of the films with a pre-peak intensity close to the spinel reference. It should be noted that pre-peaks at 7114.2 eV or higher energy levels are consistent with the presence of Fe^3+^ ions, while contributions at lower energy values of around 7112 eV are expected for Fe^2+^ ions [40]. Thus, no Fe^2+^ contribution in the octahedral sites is observed in our films.

The oxidation state of the Fe-absorbing atoms in each film has been determined by taking into account the linear relationship between the absorption edge position and the average oxidation state of atoms, using specific references [41,42,43] to corroborate the pre-peak results. Figure 5c shows the energy values in the absorption edge position for the references used, as well as the values for the as-deposited and post-annealed LFO films, identifying in all cases that the average valence is 3+ for the Fe absorbing cations. Remarkably, as in the case of stoichiometric target LiFe_5_O_8_, only Fe^3+^ cations are identified irrespective of the energy of the beam assistance for stoichiometric, lithium-rich, and lithium-deficient films, but are also identified independently of the post-annealing process. However, it has been reported that in lithium deficient films, compared to the stoichiometric phase LiFe_5_O_8_, the coexistence of Fe^2+^ and Fe^3+^ on octahedral sites that induces semiconducting behavior [17], which was not to be expected in our films.

**Figure 6:**
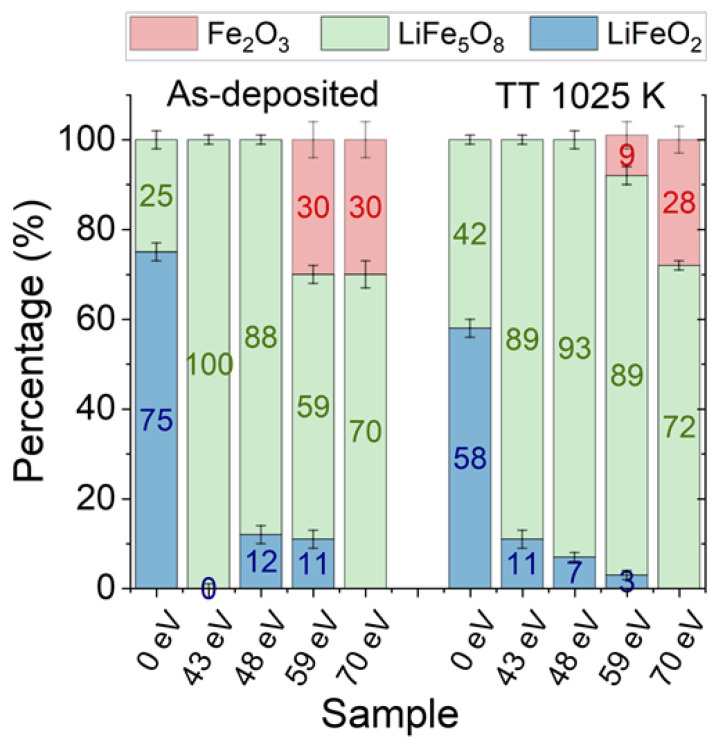
Compositional analysis of the thin films obtained from a linear combination fitting of the XANES spectra, employing LiFeO_2_, LiFe_5_O_8_, and α-Fe_2_O_3_, γ-Fe_2_O_3_, and Fe_3_O_4_ references.

A linear combination fitting of thin films was carried out in order to identify the phase composition as a function of the energy of the assisting beam and the effect of post-annealing. Figure 6 displays the results. For the as-deposited film without ion assistance, 75% of LiFeO_2_ was obtained, which was reduced to 58% after the post-annealing process at 1025 K. The secondary phase was LiFe_5_O_8_, which was in agreement with the pre-peak analysis. As beam assistance was applied during film growth for both as-deposited and post-annealed films, the predominant phase was LiFe_5_O_8_, found either as a single phase or with a low percentage of the rock-salt phase at 43 and 48 eV, and in combination with the α-Fe_2_O_3_ at 59 eV. For the highest values of energy of the assisting ion beam, i.e., 70 eV, the LiFe_5_O_8_ appeared along the α-Fe_2_O_3_ as a secondary phase and without the presence of the rock-salt phase. The presence of α-Fe_2_O_3_ at high energies of the assisting ion beam, i.e., 59 and 70 eV, was already identified by XRD, corroborating these results.

**Figure 7:**
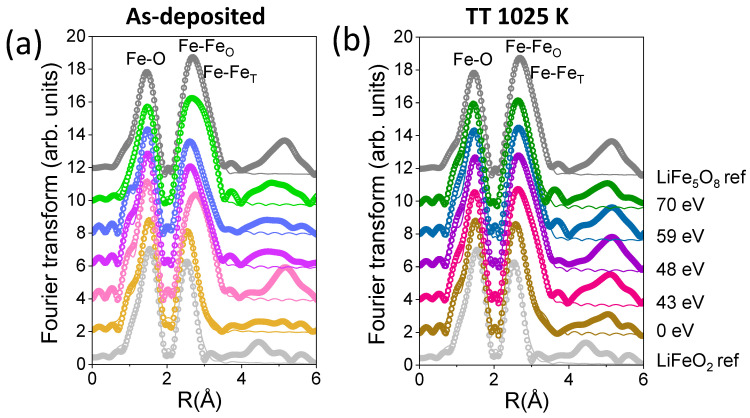
FT modulus of the EXAFS spectra at the Fe K-edge and best-fitting simulations (continuous lines) of the (**a**) as-deposited and (**b**) post-annealed LFO films as well as the spinel and rock-salt lithium ferrite references.

The short-range order around the Fe cations was analyzed as a function of the ion assistance and post-annealing process. Figure 7 displays the modulus of the Fourier transform (FT) of the EXAFS signal at the Fe K-edge for all films. FT is performed on the k^3^χ(k)-weighted EXAFS signal between 2.6 and 11.0 Å^−1^. Experimental EXAFS results were fitted in the R-space in the range of 1.0–3.5 Å, using the FEFFIT code [44]. The fitting was performed by leaving the coordination number N, the interatomic distance R, and the Debye–Waller (DW) factor σ^2^ as free parameters. For the fitting of the rock-salt structure, one shell produced by the interaction of a Fe-absorbing atom with O atoms and one shell from the interaction of a Fe-absorbing atom surrounded by Fe atoms are considered. For the spinel structure, an additional third shell related to a Fe-absorbing atom surrounded by tetrahedral Fe atoms was included in the model. Table 1 displays the numerical EXAFS results obtained from the fitting for LFO films.

In the Fe K-edge EXAFS signal of the LiFeO_2_ reference, two shells are clearly recognizable. The first shell is located around 2.0 Å and is associated with the distances of Fe from O atoms (Fe-O bonds), while the second shell around 3.0 Å corresponds to the distances between the Fe cations and Fe cations located in octahedral positions (Fe-Fe_O_). For spinel ferrite (LiFe_5_O_8_) reference, three shells are included in the model. The first shell is located around 2.0 Å, corresponding to distances between the Fe and O atoms (Fe-O bonds). The second shell around 3.0 Å is related to Fe atoms with Fe cations located in octahedral positions (Fe-Fe_O_) and the third shell around 3.5 Å, which is associated with Fe cations located in octahedral positions with Fe cations situated in tetrahedral positions (Fe_O_-Fe_T_). The existence of the third shell for the spinel ferrite corresponds with the presence of Fe atoms in tetrahedral positions, while the rock-salt Fe atoms only occupy octahedral positions [45,46], confirming the XANES results.

For the thin LFO films grown without beam assistance, a similar EXAFS signal to that of the LiFeO_2_ reference can be observed, with higher intensity in the second shell (see Table 1). The presence of any Fe-Fe_T_ distance is not identifiable, as already observed in the pre-peak analysis of the XANES spectrum. For the rest of the as-deposited films, an EXAFS signal that is more similar to that of the LiFe_5_O_8_ reference can be observed, where the three shells are modeled. The most significant changes correspond to the intensity of shells, which can be attributed to the coordination of Fe cations to O, Li, and Fe ions, according to the model results (see Table 1). It should be noted that in higher energy positions, the film deposited at 43 eV shows the most similar EXAFS signal to the LiFe_5_O_8_ reference (see Figure 7a).

As the thin LFO film grown without assistance is post-annealed at 1025 K, a third shell has to be considered in the model related to Fe-Fe_T_ distances, due to the presence of a greater amount of spinel ferrite in the films in addition to the rock-salt phase, as was identified by the compositional fitting of the XANES spectra (see Figure 7). For thin films grown under beam assistance and post-annealed at 1025 K, similar results as found without post-annealing are obtained, with the most significant changes being seen in the intensity of shells. In addition to the O and Li concentrations in the structure of thin films, in films prepared at higher energies, the variations in intensities could be related to the presence of α-Fe_2_O_3_. Significant changes in the Debye–Waller factor are not identified with the beam assistance and the post-annealing of films.

**Figure 8:**
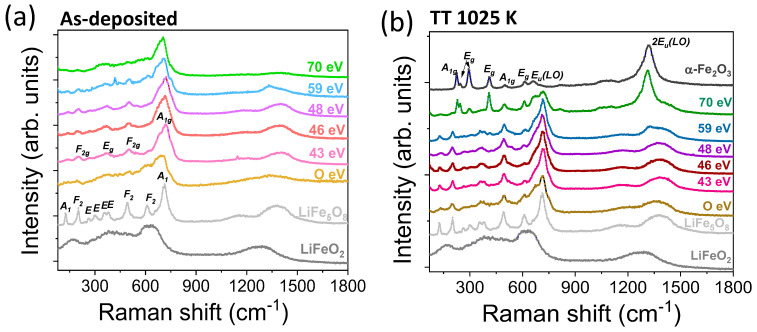
Raman spectra of the (**a**) as-deposited thin LFO films and (**b**) after a post-thermal annealing process at 1025 K. The spectra of the LiFe_5_O_8_ and LiFeO_2_ sputtering targets that are used as references, as well as those of the α-Fe_2_O_3_ reference have also been included for comparative purposes. The main Raman modes of the α and β phases of LiFe_5_O_8_ and those of α-Fe_2_O_3_ are identified on the Raman spectra of the LiFe_5_O_8_ target, for the LFO film prepared at 43 eV, and the α-Fe_2_O_3_ reference, respectively.

In a complementary way to the XAS experiments, Raman studies of the thin LFO films were conducted. According to the literature, the ordered (P4_3_32) phase of LFO shows 6A1, 14E, and 20F2 phonon modes that are allowed in the Raman spectra, while the disordered (Fd-3m) phase only has five first-order modes allowed (A_1g_, 3E_g_, and F_2g_). In addition, a strong broadening of the Raman bands of the disordered phase can be expected [47,48]. The Raman spectrum of the LiFe_5_O_8_ reference, displayed in Figure 8, is consistent with the alpha phase, showing the ten clear visible bands that are labeled in Figure 8a. In the case of the LiFeO_2_ reference, three broad bands are observed at 167, 420, and 622 cm^−1^, which is consistent with the rock-salt structure [37,49] (see the Raman bands identified in Figure 8a). The rock-salt structure exhibits weak Raman scattering and the first-order phonon modes are forbidden [37,49], but the enhancement of bands can occur due to the presence of vacancies or structural defects in the films.

The Raman spectra of the as-deposited LFO films (Figure 8a) show broad vibrational bands, with some positions near those of the LiFe_5_O_8_ reference. The as-deposited film without beam assistance exhibits a Raman spectrum that is more similar to that of the LiFeO_2_ reference, confirming the coexistence of rock-salt with the spinel ferrite on this film. Comparing the signal of this film with respect to that of the LiFeO_2_ target, narrower and more intense Raman modes are noted that can be attributed to the Li deficiency identified in the sample. The Raman spectra of the assisted films with energies between 43 and 48 eV show complex broad peaks corresponding to the A_1g_ Raman mode peaks at 719 cm^−1^, to the E_g_ Raman mode at ~370 cm^−1^, and to the F_2g_ modes at ~201, 500, and 611 cm^−1^, which could be related to a disordered phase of the spinel ferrite. However, the spectra also show small contributions at ~138, 268, and 550 cm^−1^, which may be related to some of the A_1_, E, and F_2_ phonon modes of the ordered structure [48]. For the film assisted with the highest ion energy that corresponds to the lower-lithium-content films, the spectrum only showed five broad bands at 704, 602, 493, 360, and 200 cm^−1^, due to the disordered spinel ferrite structure.

After post-thermal annealing, some characteristic bands of the α-ordered phase are clearly visible in Figure 8b, indicating that a transformation from the β to the α phase has taken place. In the sample without assistance and the ones assisted with energies between 43 and 48 eV, a more intense peak appears at 715 cm^−1^ that can be related to both the α and β phases. The peaks at ~611, 495, and 200 cm^−1^ can also be assigned to the Raman modes of the α and β phases. However, the presence of peaks at 130, 268, 380, 362, and 530 cm^−1^ clearly indicates the presence of an ordered phase. In the case of bulk crystal samples, it is well known that a transformation from the ordered to the disordered phase takes place after annealing in air followed by rapid quenching in liquid nitrogen [15,48]. However, in the thin film samples, post-annealing at 1025 K induces a disorder–order transformation, which seems to be an appropriate process for obtaining crystalline LFO films. For films deposited with assisting ion energies of 59 and 70 eV and then post-annealed, the presence of some vibrational bands related to hematite is identified by Raman spectroscopy, confirming the XRD and XAS results. Hematite exhibits seven phonon modes (2A_1g_ + 5E_g_) that are allowed, in addition to others that are not allowed, such as the infrared (IR) active longitudinal optical (LO) Eu mode around 667 cm^−1^, which is activated by disorder in the α-Fe_2_O_3_ structure, and the band around 1325 cm^−1^ that can be attributed to an overtone of the LO band [50]. The more intense ones are labeled in Figure 8b. In those films, besides the hematite overtone, the vibration modes around 230, 294, and 410 cm^−1^ are assigned to the hematite phase that is present in the films. In all cases, the Raman spectra are represented in the 80–1800 cm^−1^ Raman shift range since any Raman contribution appears above 1500 cm^−1^.

To summarize, crystalline and ordered lithium ferrite with a spinel structure is obtained after the post-annealing of the thin LFO films deposited by DIBS; however, its lattice constant increases with the energy of the assisting ion beam, thus, with lithium vacancies, keeping a spinel structure in which no change in the average valence of the Fe cations is found. In the case of thin LFO films without ion assistance, the post-annealing process at 1025 K reduces the amount of the rock-salt structure, while for the LFO films assisted with high ion beam energies, the post-annealing process induces an increase in the hematite amount. In addition, the presence of the disordered spinel ferrite phase, identified for the as-deposited films, is transformed to the ordered phase with the subsequent post-annealing process.

**Figure 9:**
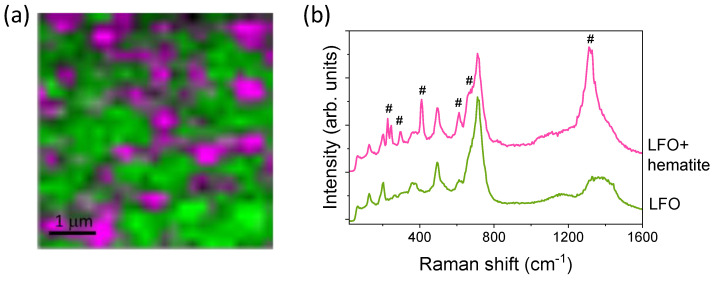
(**a**) Raman intensity mapping of a thin LFO film obtained with the assistance energy of Ar^+^ + O_2_^+^ ions of 59 eV. (**b**) Raman spectra extracted from the green and pink areas of the Raman map, corresponding to LFO and LFO + α-Fe_2_O_3_, respectively. Raman modes marked with # symbols correspond to the Raman bands of hematite.

In order to analyze the local disposition of secondary phases in the LFO films, Raman mappings on several regions of the films were performed. Figure 9a shows the intensity of Raman mapping collected on the surface of the thin film grown at 59 eV and post-annealed at 1025 K, in which the coexistence of the spinel LFO ferrite along with the hematite by XRD and XAS has already been identified (see Figure 9b). Clearly, in addition to the spinel phase distributed throughout the film, there are some local regions where the spinel ferrite coexists with the hematite phase, with sizes of between 300 nm and 1 μm. Vibrational modes at 229, 245, 296, 410, 613, 673, and 1320 cm^−1^ of hematite (marked with # in Figure 9b) are identified along with the Raman bands of the spinel ferrite. These results could indicate that the concentration of hematite is larger than that identified by XRD and XAS, but the high scattering of this Fe oxide phase in the Raman spectroscopy should be taken into account, a value that could be overestimated if compositional quantification is carried out with this experimental technique. Something similar happens to the rock salt phase, wherein the Raman scattering is very low and underestimation could occur.

## 4. Conclusions

In this work, thin lithium ferrite films on (0001) Al_2_O_3_ substrates have been grown by dual ion beam sputtering, tailoring the lithium content by using the different energies of the assisting Ar^+^ + O_2_^+^ ions. In addition to the lithium concentration, the composition and the structural characteristics are controlled by both the assisting ion energy and the post-annealed process at 1025 K. The combination of several advanced structural techniques has allowed the identification of crystalline spinel ferrite, along with long-range disorder rock-salt ferrite as the principal phase for the as-deposited film without assistance. Assistance with Ar^+^ + O_2_^+^ ions generates highly out-of-plane-oriented (111) thin LFO films. In addition, in the films assisted with high-energy ions, we can identify the presence of hematite, which is associated with the deficiency of Li cations in the structure, forming the spinel ferrite. In addition, the identification of a disordered spinel structure has been possible in the as-grown samples as well as the disorder–order transformation of the spinel ferrite with post-annealing at 1025 K. With the higher assisting beam energies, as the Li concentration decreases, the hematite phase emerges in coexistence with the spinel ferrite.

Therefore, we demonstrate that dual ion beam sputtering is a suitable technique, not only to allow the growth of stoichiometric and highly (111) oriented thin LFO films but also to control the amount of Li and a structure in both shorter and larger orders, allowing the control of the functional properties of the films for the development of future spintronic devices. The composition and energy of not only the assisting ions but also the post-annealing processes are relevant for tailoring the structure and composition of thin LFO films.

## Figures and Tables

**Table 1:** Results of the EXAFS fittings of Li ferrites at the Fe K-absorption edges for each shell. N: coordination number, R: interatomic distance, and DW: Debye–Waller factor.

Sample	Shell	N	R (Å)	DW (Å^2^)
**LiFeO_2_ reference**	Fe-O	6	2.002 (2)	0.008 (2)
	Fe-Fe	4	3.001 (7)	0.012 (2)
**LiFe_5_O_8_ reference**	Fe-O	5.2	1.96 (1)	0.010 (1)
	Fe-Fe	4	3.02 (2)	0.013 (4)
	Fe-Fe	6	3.48 (1)	0.016 (5)
**LFO 0 eV**	Fe-O	5.85 (7)	1.998 (5)	0.008 (1)
	Fe-Fe	5.1 (3)	3.016 (5)	0.014 (2)
**LFO 43 eV**	Fe-O	4.9 (9)	1.954 (6)	0.008 (2)
	Fe-Fe	2.4 (4)	3.01 (2)	0.009 (4)
	Fe-Fe	4.1 (5)	3.48 (1)	0.011 (5)
**LFO 48 eV**	Fe-O	5.07 (8)	1.965 (5)	0.008 (2)
	Fe-Fe	3.6 (5)	3.00 (1)	0.012 (4)
	Fe-Fe	2.9 (5)	3.47 (6)	0.012 (6)
**LFO 59 eV**	Fe-O	5.0 (8)	1.974 (5)	0.009 (2)
	Fe-Fe	4.1 (5)	3.00 (1)	0.014 (4)
	Fe-Fe	2.6 (4)	3.47 (2)	0.012 (6)
**LFO 70 eV**	Fe-O	4.7 (1)	1.969 (6)	0.009 (2)
	Fe-Fe	4.7 (5)	3.01 (1)	0.014 (4)
	Fe-Fe	4.3 (4)	3.483 (9)	0.011 (4)
**LFO 0 eV 1025 K**	Fe-O	5.1 (9)	1.987 (6)	0.008 (2)
	Fe-Fe	3.8 (4)	3.00 (1)	0.010 (3)
	Fe-Fe	2.2 (2)	3.50 (2)	0.013 (1)
**LFO 43 eV 1025 K**	Fe-O	5.1 (1)	1.965 (6)	0.009 (2)
	Fe-Fe	3.4 (4)	3.00 (2)	0.011 (4)
	Fe-Fe	4.2 (5)	3.48 (1)	0.012 (5)
**LFO 48 eV 1025 K**	Fe-O	5.2 (1)	1.965 (6)	0.009 (2)
	Fe-Fe	3.2 (4)	3.01 (1)	0.010 (4)
	Fe-Fe	4.6 (5)	3.49 (1)	0.013 (5)
**LFO 59 eV 1025 K**	Fe-O	5.0 (1)	1.962 (8)	0.009 (2)
	Fe-Fe	3.4 (7)	3.01 (3)	0.011 (6)
	Fe-Fe	4.7 (9)	3.48 (2)	0.015 (9)
**LFO 70 eV 1025 K**	Fe-O	5.6 (1)	1.973 (4)	0.011 (3)
	Fe-Fe	5.0 (8)	3.01 (3)	0.014 (7)
	Fe-Fe	4.2 (6)	3.46 (4)	0.018 (8)

## Data Availability

Data will be made available upon reasonable request.
